# Application of prophage sequence analysis to investigate a disease outbreak involving *Salmonella* Adjame, a rare serovar and implications for the population structure

**DOI:** 10.3389/fmicb.2023.1086198

**Published:** 2023-03-03

**Authors:** Ruimin Gao, Marc-Olivier Duceppe, Marie Anne Chattaway, Lawrence Goodridge, Dele Ogunremi

**Affiliations:** ^1^Ottawa Laboratory Fallowfield, Canadian Food Inspection Agency, Ottawa, ON, Canada; ^2^Department of Food Science and Agricultural Chemistry, McGill University, Ste Anne de Bellevue, QC, Canada; ^3^Gastrointestinal Bacteria Reference Unit, United Kingdom Health Security Agency, London, United Kingdom; ^4^Department of Food Science, Canadian Research Institute for Food Safety, University of Guelph, Guelph, ON, Canada

**Keywords:** *Salmonella*, Adjame, prophage, foodborne outbreak, SNP, heterogeneity, discriminative, diversity

## Abstract

**Introduction:**

Outbreak investigation of foodborne salmonellosis is hindered when the food source is contaminated by multiple strains of *Salmonella*, creating difficulties matching an incriminated organism recovered from patients with the specific strain in the suspect food. An outbreak of the rare *Salmonella* Adjame was caused by multiple strains of the organism as revealed by single-nucleotide polymorphism (SNP) variation. The use of highly discriminatory prophage analysis to characterize strains of *Salmonella* should enable a more precise strain characterization and aid the investigation of foodborne salmonellosis.

**Methods:**

We have carried out genomic analysis of *S.* Adjame strains recovered during the course of a recent outbreak and compared them with other strains of the organism (*n* = 38 strains), using SNPs to evaluate strain differences present in the core genome, and prophage sequence typing (PST) to evaluate the accessory genome. Phylogenetic analyses were performed using both total prophage content and conserved prophages.

**Results:**

The PST analysis of the *S.* Adjame isolates showed a high degree of strain heterogeneity. We observed small clusters made up of 2-6 isolates (*n* = 27) and singletons (*n* = 11) in stark contrast with the three clusters observed by SNP analysis. In total, we detected 24 prophages of which only four were highly prevalent, namely: Entero_p88 (36/38 strains), Salmon_SEN34 (35/38 strains), Burkho_phiE255 (33/38 strains) and Edward_GF (28/38 strains). Despite the marked strain diversity seen with prophage analysis, the distribution of the four most common prophages matched the clustering observed using core genome.

**Discussion:**

Mutations in the core and accessory genomes of *S.* Adjame have shed light on the evolutionary relationships among the Adjame strains and demonstrated a convergence of the variations observed in both fractions of the genome. We conclude that core and accessory genomes analyses should be adopted in foodborne bacteria outbreak investigations to provide a more accurate strain description and facilitate reliable matching of isolates from patients and incriminated food sources. The outcomes should translate to a better understanding of the microbial population structure and an 46 improved source attribution in foodborne illnesses.

## Introduction

The bacterial genus *Salmonella* consists of organisms responsible for substantial human and livestock morbidity and mortality by causing a spectrum of diseases manifesting as enteric fever, septicemia, and gastroenteritis (Dekker and Frank, 2015). *Salmonella enterica* is the main species of medical and veterinary importance and consists of about 2,500 serovars. Based on their degree of host adaptability in both humans and animals, *Salmonella* are classified into two groups (Gal-Mor et al., 2014; Jajere, 2019). The serovars adapted to specific hosts typically cause systemic, typhoid-like symptoms, e.g., Typhi, Paratyphi, Gallinarum, Abortusovis, and Pullorum, and are distinct in behavior from the generalists which can infect many hosts. The generalists cause only a limited pathology, most commonly a gastroenteritis, and are referred to as non-typhoidal *Salmonella* (NTS), e.g., Typhimurium and Enteritidis. The NTS represent a major cause of diarrhea globally (Cheng et al., 2019). Although only a small proportion of the *Salmonella* serovars have been associated with foodborne outbreaks (Khakhria et al., 1997; Centers for Disease Control and Prevention, 2016; European Food Safety Authority, 2022), a single food source may be contaminated with multiple serovars or multiple strains of the same serovar (Whitney et al., 2021; Schwensohn et al., 2022), which makes identifying the source of an infection very difficult and continues to hinder the control of human salmonellosis.

Whole-genome sequencing (WGS) is now the main approach for characterizing isolates of *Salmonella* for outbreak investigation and surveillance and has almost completely replaced the traditional phenotypic and molecular typing tools such as serotyping, pulsed-field gel electrophoresis (PFGE), and phage typing (PT). Determining the serotype or serovar is desired because of its relevance to *Salmonella* epidemiology, and the serological agglutination procedure traditionally used is now mostly replaced with bioinformatics analysis of sequence reads as performed by means of two main algorithms, namely, the Salmonella *In Silico* Typing Resource (SISTR) software (Yoshida et al., 2016) and the SeqSero2 software (Zhang et al., 2015). Delineating strains of the same serovar was traditionally achieved using the PFGE and PT methods but is now accomplished by one of two approaches. The first approach is to determine the sequence type of the organism and is commonly achieved using the multilocus sequence typing (MLST) method which was originally designed to evaluate nucleotide sequences of five to seven housekeeping genes of the organism (Maiden et al., 1998). This procedure can now be more easily performed, e.g., by uploading the genome reads into the web-based EnteroBase platform to provide multiple, hierarchical levels of characterization and strain differentiation (Zhou et al., 2020). The second approach for strain differentiation relies on single-nucleotide polymorphism at variable sites in the genome. Many algorithms have been developed for the purpose of characterizing SNPs in either the core genome or the pan-genome. The SNVPhyl algorithm developed by the Public Health Agency of Canada identifies high-quality SNPs among a set of selected isolates and is useful for generating phylogenetic trees from these SNPs (Petkau et al., 2017). A similar algorithm, the SnapperDB which was developed by the United Kingdom Health Security Agency (UKHSA) also identifies high-quality SNPs useful for evaluating genetic distances among the genomes and for inferring relatedness among strains (Dallman et al., 2018). Parsnp is yet another algorithm which detects core-genome SNP in bacterial genomes and with the aid of an adjunct interactive tool known as Gingr, displays informative overviews for specific sub-clades and genomic regions (Treangen et al., 2014). The kSNP tool detects SNPs in the pan-genome but is uniquely able to carry out comparisons among genomes without a requirement for genome alignment or a reference genome (Gardner et al., 2015).

WGS tools were applied to investigate an outbreak of *Salmonella enterica* serovar Adjame in England in 2017. The organism is a rare NTS for which the first clinical case was reported in Cote d’Ivoire in 1967 (Le Minor et al., 1967). The first documented clinical case in the western world was observed in the United Kingdom in 1993 and was followed by 13 sporadic cases leading up to the year 2016 (Chattaway et al., 2019). An epidemiological investigation of the latest outbreak identified herbs and spices as the most likely food vehicles (Chattaway et al., 2019), and WGS analysis using both EnteroBase *Salmonella* core-genome MLST (cgMLST, allele-based method^1^) and UKHSA SnapperDB pipeline (Dallman et al., 2018) showed the presence of heterogeneous strains which were grouped into two clusters (Chattaway et al., 2019). The two outbreak clusters were shown to be distinct from a third cluster comprised of *S*. Adjame strains from sporadic cases present in the UKHSA archive. Strain characterization and identification of multiple strains relied on core-genome analysis but without a good understanding of the population structure of the rare organism, the question arises whether the strain characterization was sufficiently rigorous to provide information that may facilitate an intervention to forestall a spread of a current outbreak or combat a future outbreak. This prompted us to investigate the accessory genome of isolates of *S*. Adjame recovered before, during, or around the time of the 2017 England outbreak to further characterize the population and ask whether the analysis could lead to a more defined clustering or alternatively confirmed the heterogeneity of strains involved.

A major contributor to the accessory genome of the *Salmonella* organism are prophages or bacteriophages which have become integrated into the bacterial chromosome (Ogunremi et al., 2014). Other contributors to the accessory genome include a myriad of genetic sequences that enter the bacteria cell by horizontal gene transfer and may end up in the chromosome or in a plasmid and includes the following: antibiotic resistance genes, insertion sequences, genomic islands, integrative conjugative elements, and other transposons (Subedi et al., 2019). Bacterial plasmids as an entity also meet the definition for inclusion in the accessory genome since they are not always present in all or a majority of the strains of a species. Bacteriophages are considered the most abundant “life form” on the planet (Wommack and Colwell, 2000), representing an incredibly diverse gene pool, and have been shown to contribute significantly to host bacterial evolution (Fortier and Sekulovic, 2013). As part of their parasitic life cycle in bacteria, bacteriophages can either be virulent, and follow a lytic life cycle or can integrate into unique attachments sites in the chromosome of the bacteria by following the lysogenic cycle in which case they are referred to as prophages (Kropinski et al., 2007; Switt et al., 2015). Prophages have proven to be suitable markers for differentiating *S. enterica* subtypes because of the large but stable variation present in phages (Gao et al., 2020). This variation was exploited to develop a highly discriminatory prophage subtyping tool (PST) which not only distinguished serovars but possessed a very high resolution that is useful for defining epidemiologically unrelated *Salmonella enterica* serovar Enteritidis strains during foodborne outbreaks (Mottawea et al., 2018). The discriminatory ability of the method is based on variations in the composition of prophages among strains of the same organism as well as size and nucleotide differences that may occur in the same prophage in the different strains (Mottawea et al., 2018). Additional observations from the study showed that sufficient diversity in a single phage genome, namely RE-2010 phage has enough variability for distinguishing among *Salmonella* Enteritidis strains from different outbreaks (Mottawea et al., 2018). Furthermore, Goodridge and colleagues demonstrated a similar discriminatory capacity in the tyrosine integrase gene of 32 enteric prophages for the strain characterization of members of *S. enterica* (Colavecchio et al., 2017).

In the current study, we used the PST pipeline to analyze 38 strains of *S*. Adjame which represents the entire population of the organism with genome sequences available in the GenBank at the initiation of this study, providing an opportunity to characterize the strains by focusing on the accessory genome to evaluate differences among strains and to compare outbreak and sporadic strains. The study has provided an early understanding of the genomic diversity and population structure of the rare *S*. Adjame based on the available genome resources available, albeit limited. An application of core and accessory genomes analytical tools for future *S*. Adjame strains, either from outbreaks or sporadic sources should help to accurately define relatedness to hitherto observed strains and possibly facilitate a decision in support of an early intervention to prevent a developing outbreak which could have been dismissed in the absence of any knowledge of the genomic structure of the population.

## Materials and methods

### Bacterial genome sequences used in this study

The genome sequences for all the *S*. Adjame strains publicly available in the GenBank as of 30 December 2020 were retrieved and analyzed in this study (n = 38). The outbreak strains were included in the 28 strains from England, 2 strains were from Ireland, and 1 strain from Denmark, previously described in an *S*. Adjame outbreak (Chattaway et al., 2019). An additional 6 *S*. Adjame strains reported by UKHSA between 2008 and 2016, included in the report of the outbreak (Chattaway et al., 2019), were also analyzed in this study. The final strain was recovered after the year of the outbreak in 2018. Genome sequences of all the 38 isolates were retrieved from the NCBI databases using the keywords “*Salmonella* Adjame or *S*. Adjame” and consisted of Illumina paired-end sequences, 2 × 100 bp, and their related metadata from the Sequence Read Archives (SRA) as summarized in Table 1. The raw reads were trimmed to obtain a minimum PHRED quality score of Q30 from the 3′ end using the BBDuk tool (https://jgi.doe.gov/data-and-tools/bbtools/). Any trimmed read shorter than 64 bases was discarded and all reads passing quality assurance were merged using BBMerge (https://jgi.doe.gov/data-and-tools/bbtools/). The SPAdes genome assembler 3.10.1 (Bankevich et al., 2012) was used to perform *de novo* genome assembly and the quality of assembled genomes was evaluated with the QUAST software (Gurevich et al., 2013). In addition, a total of 9,066 *Salmonella enterica* genomes and the *S*. Adjame population were downloaded from the NCBI RefSeq database and used to construct a phylogenetic tree to establish its relatedness with other *Salmonella* serovars.

**Table 1:** Metadata for Analyzed 38 *Salmonella* Adjame strains from humans.

**SRA**	**Accession**	**Strain**	**Location**	**Collection date**
ERR2071995	SAMEA104196846	MS170178	Ireland	2017-June
SRR6191319	SAMN07812470	399,284	England	2017-June
SRR6191533	SAMN07812687	387,215	England	2017-June
SRR6233939	SAMN07946534	435,414	England	2017-June
SRR6234003	SAMN07946601	387,511	England	2017-June
SRR6237100	SAMN07956666	387,507	England	2017-June
SRR6190984	SAMN07812157	388,789	England	2017-July
SRR6190990	SAMN07812164	400,321	England	2017-August
SRR6191331	SAMN07812472	381,330	England	2017-June
SRR6191380	SAMN07812632	388,665	England	2017-July
SRR6192965	SAMN07816135	387,049	England	2017-July
SRR6193034	SAMN07816233	389,724	England	2017-July
SRR6193063	SAMN07816315	385,774	England	2017-June
SRR6233881	SAMN07946475	387,137	England	2017-June
SRR5583198	SAMN07152399	367,320	England	2017-May
SRR5632247	SAMN07180309	356,310	England	2017-March
SRR5632905	SAMN07180523	355,050	England	2017-March
SRR6191105	SAMN07812257	353,868	England	2017-March
SRR6191118	SAMN07812315	357,971	England	2017-March
ERR2071997	SAMEA104196848	MS170185	Ireland	2017-June
ERR2234457	SAMEA104453020	SSI-AC209	Denmark	2017-June
SRR5585224	SAMN07155888	353,918	England	2017-March
SRR6190514	SAMN07812109	383,962	England	2017-June
SRR6190534	SAMN07812124	374,589	England	2017-May
SRR6191144	SAMN07812369	409,960	England	2008-October
SRR6191363	SAMN07812500	409,961	England	2011-June
SRR6192997	SAMN07816172	416,016	England	2016-July
SRR6233875	SAMN07946470	389,598	England	2017-July
SRR6237059	SAMN07956628	388,695	England	2017-July
SRR6237069	SAMN07956644	434,304	England	2017-June
SRR6237093	SAMN07956649	408,388	England	2017-July
SRR6237095	SAMN07956662	409,962	England	2012-March
SRR6466751	SAMN08358027	411,501	England	2011-March
SRR6466752	SAMN08358026	411,502	England	2013-Feburary
SRR7351460	SAMN09434937	534,836	England	2018-March
SRR7426886	SAMN09484455	416,020	England	2017-March
SRR7516694	SAMN09652021	441,469	England	2017-November
SRR7533234	SAMN09683584	456,193	England	2017-December

### Core-genome SNP analysis by Parsnp

A core-genome-based SNP tree was constructed using Parsnp in the Harvest suite which combined the advantages of whole-genome alignment and mapped reads to produce a rapid and simultaneous analysis of thousands of microbial strains (Treangen et al., 2014). Following the alignment of the 38 *S*. Adjame sequence contigs, with the aid of the AutoRecruit feature to complete the process with the use of a reference genome, the results of the multiple alignments are displayed using the Gingr component of the Parsnp. All the 38 genome reads used as the input file were in a fasta format and set up in a specified directory, whereas the generated output files consisted of the core-genome alignments, variant call matrix, and a SNP phylogenetic tree.

### Pan-genome SNP analysis by kSNP

The collections of SNPs present in both the core and accessory genomes were analyzed using kSNP version 3.0 (Gardner et al., 2015). *De novo* assembled contigs from each of the 38 *S*. Adjame strains were used as inputs for kSNP to generate the pan-genome-based SNP analysis. The outputs generated included all the identified SNPs and a consensus of the most parsimonious phylogenetic tree.

### Prophage sequences detection and their genomic diversity analysis

The prophage content of each of the 38 *Salmonella* Adjame strains was analyzed using our previously described PST procedure (Mottawea et al., 2018). Briefly, phage sequences present in contigs of 2,000 bp and bigger were detected using the PHASTER software (Arndt et al., 2016). The clustering of all identified phage sequences was performed using CD-HIT-EST to produce a prophage matrix table (Fu et al., 2012), which was fed into the Quantitative Insights Into Microbial Ecology (QIIME) software to generate a prophage phylogenetic tree (Caporaso et al., 2010). In addition, the four most prevalent individual prophages present among the 38 strains were separately characterized by identifying conserved regions using sequences alignment features, as described below.

### Phylogenetic analysis of the common *Salmonella* Adjame prophages

Prophage sequences detected by PHASTER and found to be conserved within the population were excised and saved as fasta files and aligned with one another using the Clone Manager software (version 9, Scientific and Educational software, Westminister, CO). Multi-way DNA alignment was carried out, with the exhaustive pairwise alignments of all sequences with gaps, using as scoring matrix parameters: Mismatch 2, OpenGap 4, and ExtGap 1. Phylogenetic analysis was carried by means of Neighbor-Joining method. In strains containing all 4 conserved prophages, the sequences were concatenated to form one DNA fragment and used to perform similar multi-DNA alignment as described above, and for constructing a phylogenetic tree.

### Evolutionary relatedness analysis using in-house pipeline genome comparator

To evaluate the relatedness of the rare *Salmonella* Adjame to other serovars within the genus *Salmonella,* 9,066 *Salmonella enterica* genomes including the 38 strains of *S*. Adjame strains, were downloaded from the NCBI and used to construct a Neighbor-Joining phylogenetic tree accomplished with an in-house rapid script named genome-comparator.^2^

## Results

### Genomes of *Salmonella* Adjame

The assembled *S*. Adjame genomes showed very good quality based on QUAST analysis, as shown in Supplementary Table 1. There were 30 to 50 contigs for each of the assembled genomes except for one strain that had 311 contigs (i.e., SRA Accession number SRR6190514). The total length of the genomes ranged from 4,398,656 bp to 4,741,330 bp and the average genome length was 4,612,194 bp (n = 38 strains). In addition, the GC content ranged from 52.0 to 52.9%, with an average of 52.3%. The N50 for all the 38 genome assemblies ranged from 177,929 bp to 529,036 bp with an average of 298,565 bp.

### Core-genome analysis of *Salmonella* Adjame genomes using the rapid Core-genome multi-alignment tool Parsnp

**Figure 1:**
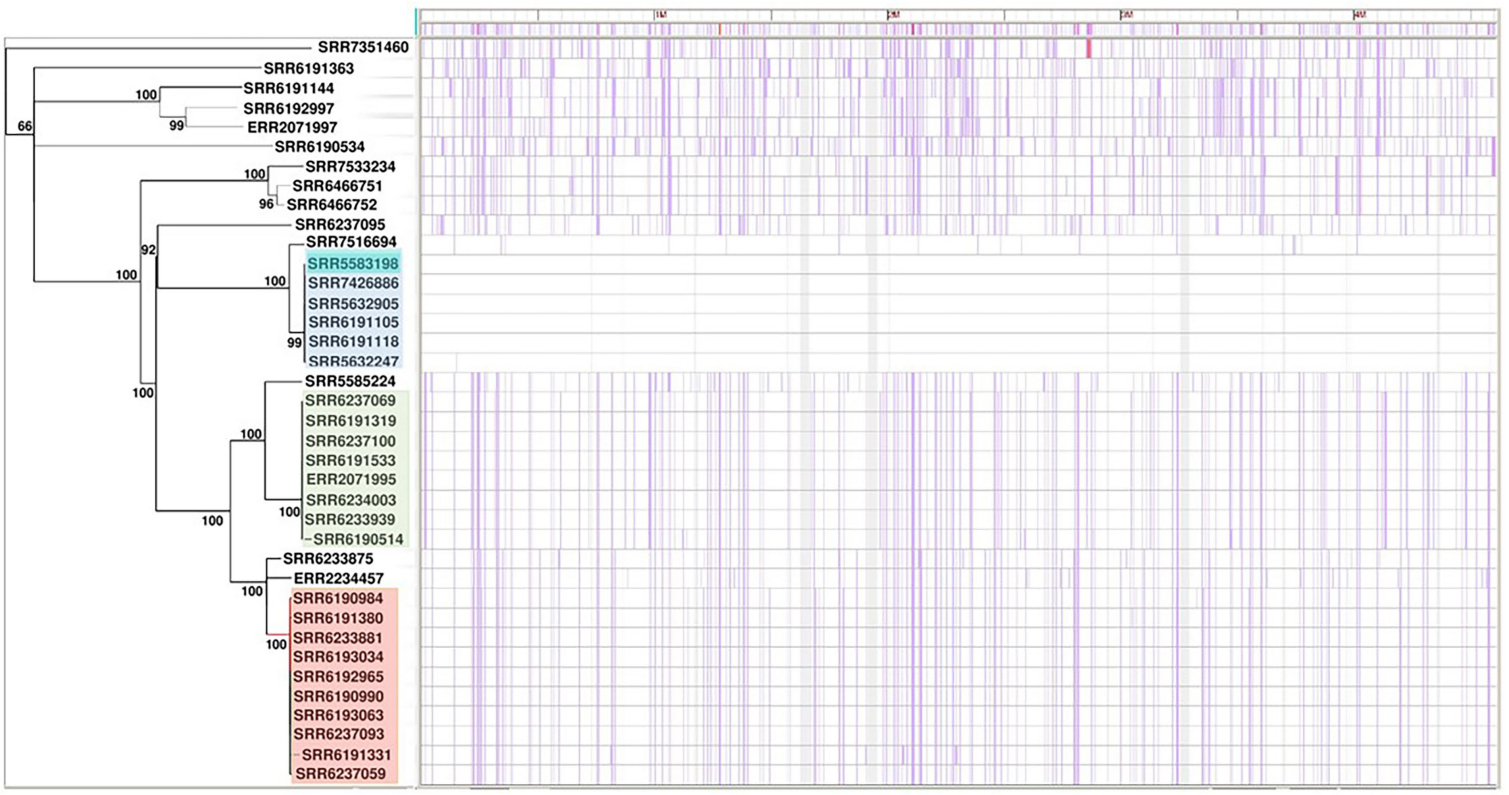
Core-genome-based phylogenetic tree of single-nucleotide polymorphism (SNP) of *Salmonella* Adjame strains (*n* = 38). The one cluster highlighted in blue represented strains recovered in patients in the United Kingdom in March 2017. The clusters highlighted in green and red are parts of the June–July 2017 outbreak. The purple vertical lines represent detected SNPs among all the studied strains with strain SRR5583198 reference (dark blue color highlighted within the blue cluster).

The phylogenetic analysis of the 38 *S*. Adjame genomes based on SNP data as generated by the Parsnp software showed three distinct clusters of strains in addition to other strains that either clustered loosely or failed to cluster (Figure 1). The three tight clusters accurately mirrored the observations by Chattaway et al. (2019), in which two of the clusters were involved in the 2017 outbreak. Consequently, we have used the same color coding in our illustration: cluster 1 (blue) consisted of six strains all isolated before the outbreak and made up of a new member in the collection (SRR7426886) and five previously identified strains; cluster 2 (green) consisted of a total of eight strains two of which are new members (SRR6190514 and SRR6237069) and six previously identified strains of which five were involved in the outbreak; and cluster 3 (red) consisted of eight outbreak strains and two new members (SRR6237059 and SRR6237093). In contrast to the observation by Chattaway et al. (2019) however, we observed that the green and red clusters which contained the 14 outbreak strains shared a more common ancestor (Figure 1), whereas a more recent ancestor was observed for the blue and red clusters in the previous report. We observed that four of the seven strains not analyzed by Chattaway et al. (2019) were distributed into the previously defined red (n = 2), green (n = 1) and blue (n = 1) clusters, while the remaining three isolates did not cluster with any group.

### Pan-genome analysis for *Salmonella* Adjame using the kSNP tool

**Figure 2:**
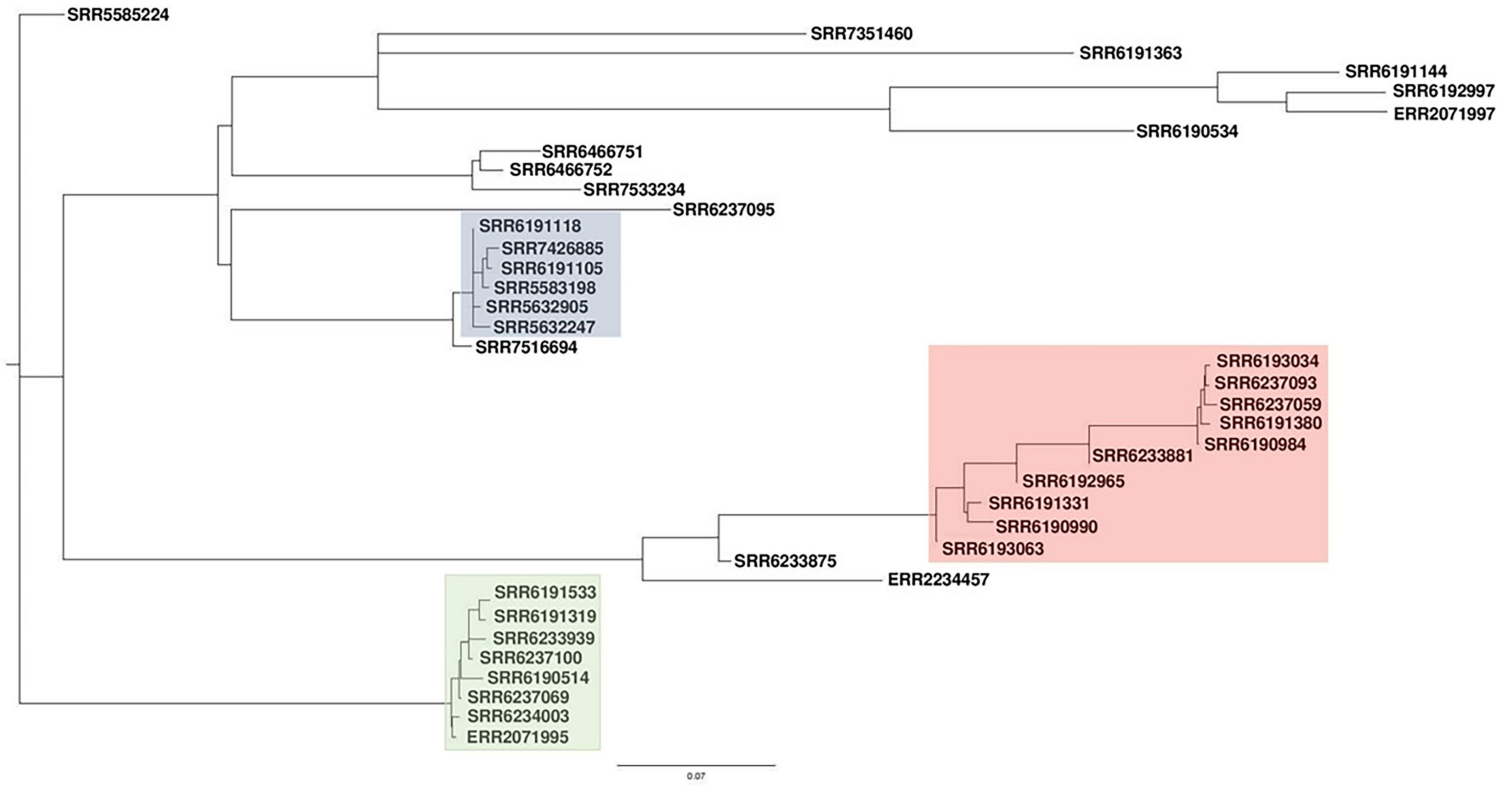
Pan-genome-based parsimony tree of single-nucleotide polymorphism (SNP) of *Salmonella* Adjame strains (*n* = 38). The tree was generated using the program named kSNP. The blue cluster represented strains obtained from patients in the United Kingdom in March 2017. The clusters highlighted as green and red represented some of the strains recovered from patients in the United Kingdom during the June–July 2017 outbreak.

Since the inclusion of the accessory genomes have in the past illuminated our investigation of subtype differences among *Salmonella* strains in the same serovar., we were interested to know whether a SNP analysis of the entire genome including the accessory fraction would prove to be more informative than a core SNP analysis. To that end, we carried out the pan-genome-based kSNP analysis on all the strains and found an identical pattern of phylogenetic clustering in that all the three clusters (blue, red, and green) and the less clustered and singletons were observed (Figure 2), similar to that of the core-genome SNP tree (Figure 1). The clusters observed by the kSNP analysis, which is similar to the Parsnp, were also comparable to the cgMLST phylogenetic tree and SNP grape tree by Chattaway et al. (2019). Members of the three kSNP clusters were however more noticeably disparate as demonstrated by the presence of many, albeit short branches in the colored kSNP clusters, when compared with the output from core SNP analysis which resulted in tight clusters. We also noticed a closer relationship between the red and blue clusters in contrast with the Parsnp analysis.

### Prophage diversity of *Salmonella* Adjame

**Figure 3:**
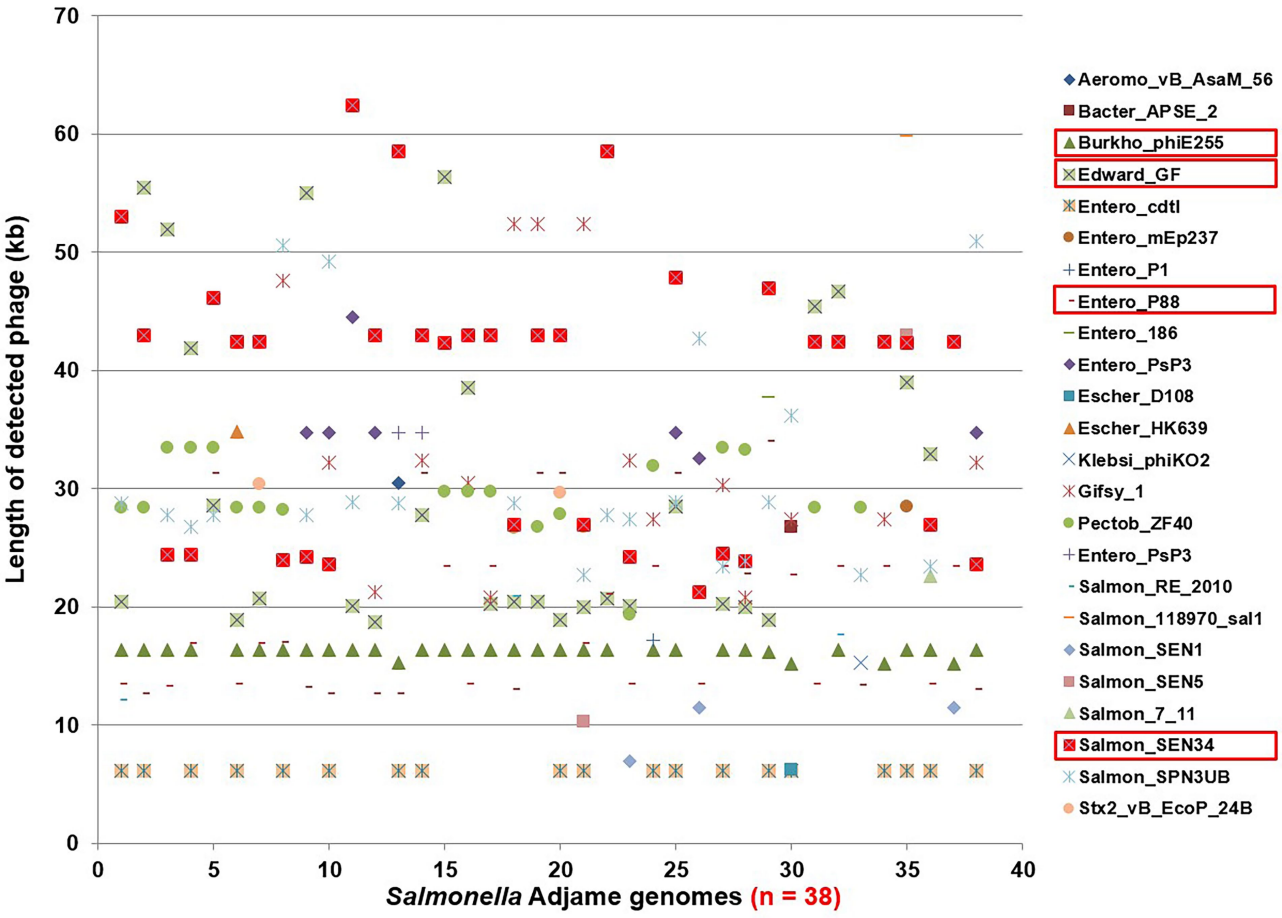
Size distribution of prophages found in *Salmonella* Adjame strains (n = 38) as detected by PHASTER. The x axis represents each strain in this study, and the y axis represents the length of 24 detected prophages in all *S*. Adjame strains.

The contrast between the tight clusters seen with the Parsnp analysis (Figure 1) and the intra-cluster branching patterns observed following the kSNP analyses, with the latter likely being a consequence of the inclusion of the accessory genome in the kSNP analysis, led us to evaluate *S*. Adjame prophage content which we previously showed to be the major component of the accessory genome in the *Salmonella* chromosome (Ogunremi et al., 2014). A total of 24 different prophages were detected among the 38 genomes of *Salmonella* Adjame using PHASTER. We also observed that a prophage may show a considerable size variation among different Adjame strains as shown in Figure 3. Generally, the overall length of all the detected prophages ranged from 6 kb to 72 kb. The four most common prophages were Entero_p88, Salmon_SEN34, Burkho_phiE255, and Edward_GF (highlighted as red boxes in Figure 3). Entero_p88 was the most prevalent and was found in 36 out of 38 strains. The sizes of the Entero_p88 prophage showed a large range from 12,704 bp to 34,000 bp.

### Prophage typing reveals marked heterogeneity among *Salmonella* Adjame strains

**Figure 4:**
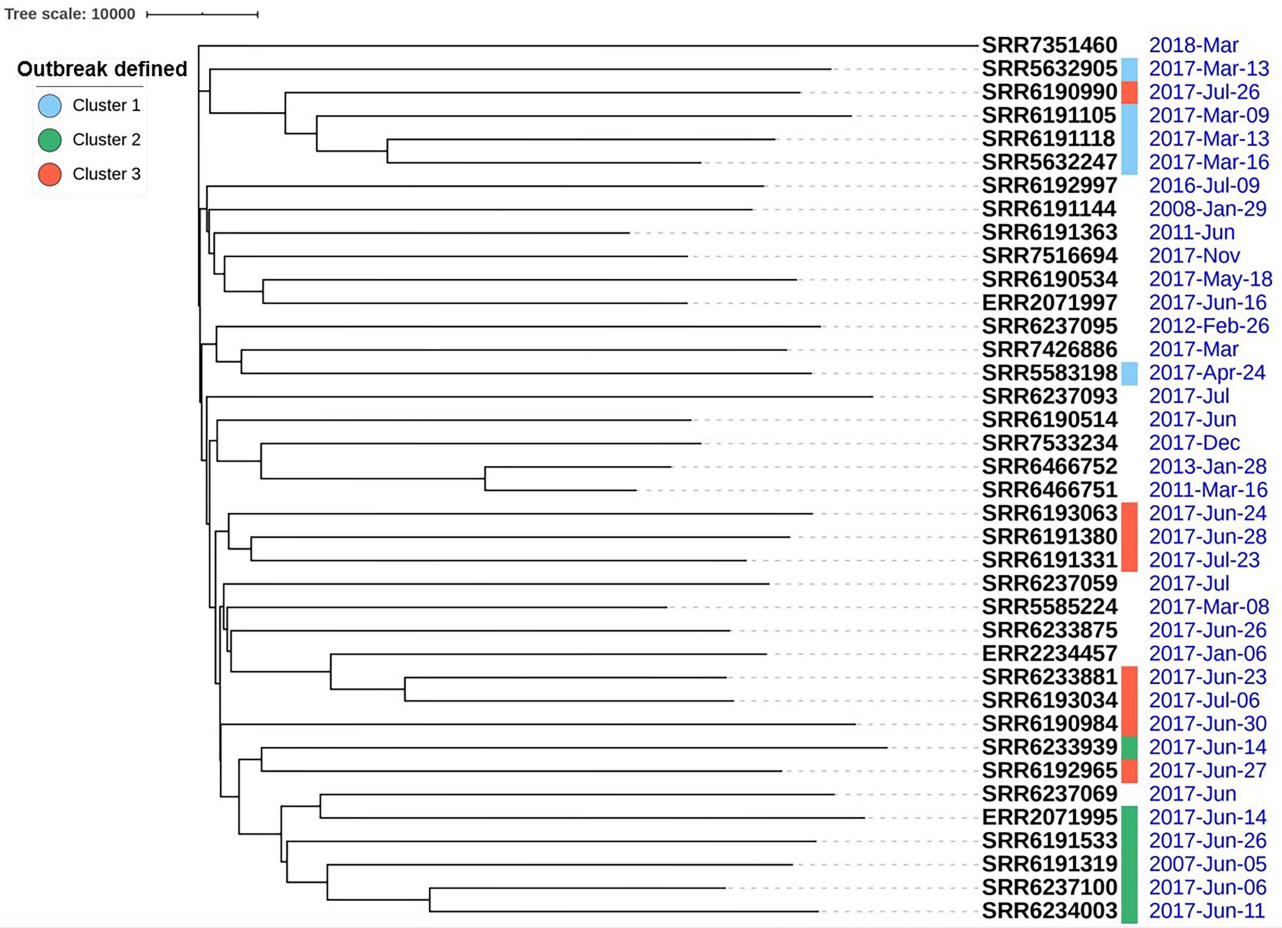
High heterogeneity of 38 *Salmonella* Adjame strains shown with prophage sequence typing pipeline analysis. Bray-Curtis distances among 38 strains are calculated based on prophage sequence CD-HIT-EST clustering parameters of sequence identity cut-off of 100% and minimal length similarity of 99%.

We applied our previously described PST pipeline to the assembled *S*. Adjame genomes and was able to demonstrate a very marked heterogeneity among the strains (Figure 4), more pronounced than that reported following cgMLST and SnapperDB SNP analyses (Chattaway et al., 2019). No two strains had an identical complement of prophages based on nucleotide composition and size, and this is reflected in the lack of strain clustering in the phylogenetic tree. Nevertheless, degrees of varying relatedness were still noticeable among some strains. For instance, five of the six outbreak strains belonging to SNP cluster 2 (blue cluster) showed more relatedness to one another by the PST than with the remainder of the collection (Figure 4). While the red and green clusters shared a common ancestor based on the core-genome analysis using Parsnp (Figure 1), no such degree of relatedness was observed among the constituent strains by means of the PST analysis (Figure 4). Furthermore, one strain (SRR6190990) from the red cluster showed a degree of relatedness with four strains that belonged to the blue cluster; and one strain (SRR6233939) from the green cluster had a prophage complement closest to other strains belonging to the red cluster.

### Phylogenetic analysis of *Salmonella* Adjame based on the conserved Entero_p88 prophage

**Figure 5:**
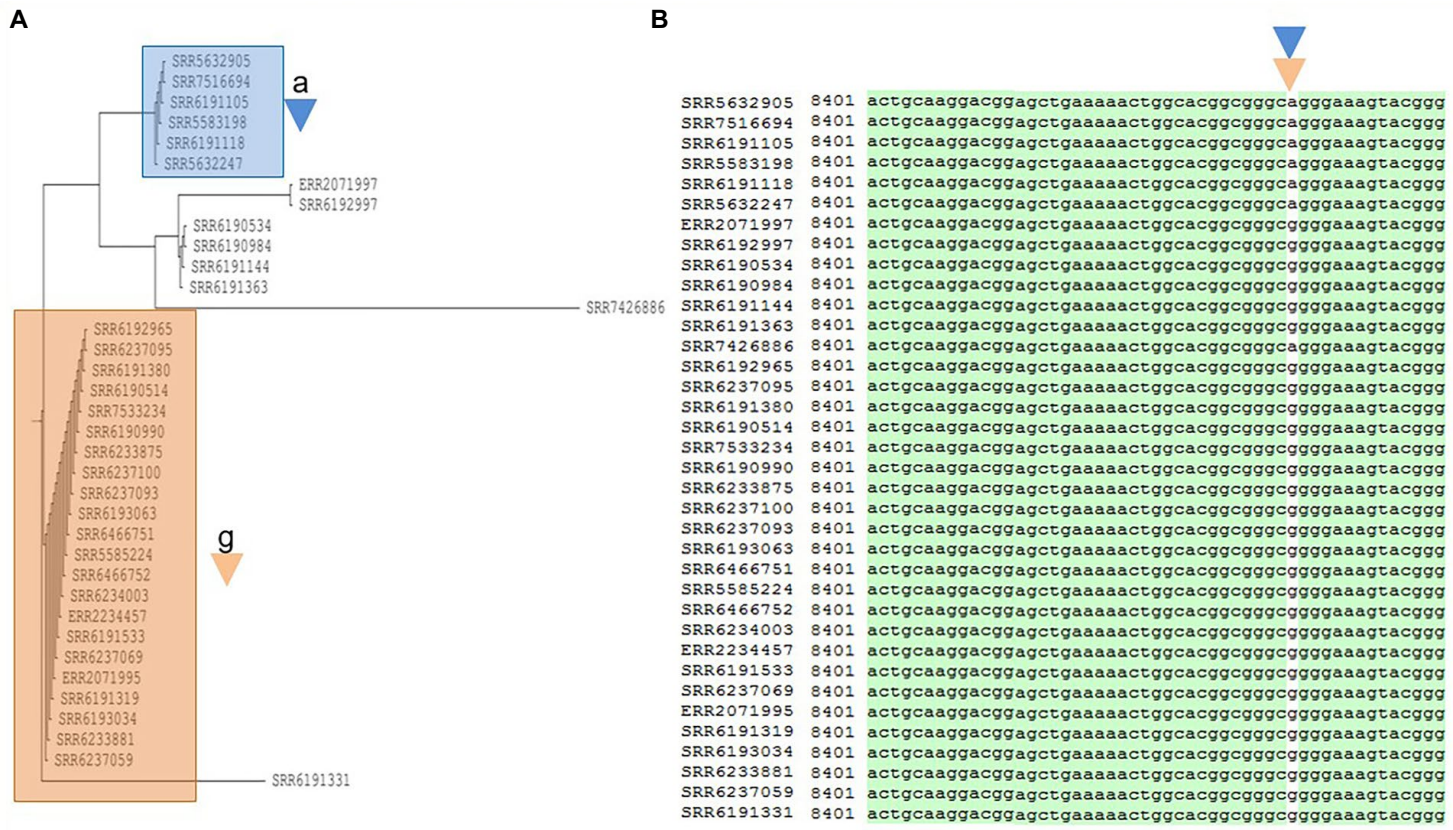
Phylogenetic tree built for 36 *Salmonella* Adjame strains which contains the most common prophage named Entero_p88 **(A)**. The detected variants are shown in white block **(B)**, with the nucleotide “a” (blue triangle) being detected in the blue cluster, and the nucleotide “g” (brown triangle) being detected in the brown cluster by merging both the green and red clusters from the June–July 2017 outbreak.

The heterogeneity seen at the prophage level suggests that *S*. Adjame may be prone to divergent evolution; nevertheless, the considerable conservation observed among many of the prophages provides an opportunity to explore commonality of the accessory genome and provide insights into the population structure of the rare organism. Given the wide size range of the prophage Entero_p88 (12,704 bp to 34,000 bp), the most conserved region was extracted among all the sequences using the multi-way DNA alignment (Clone Manager version 9, Cary, North Carolina). The total length of all the aligned sequences was 12,704 bp (Supplementary Figure 1A) and a Neighbor-Joining phylogenetic tree was constructed with two main clusters observed (Figure 5A). The first cluster consisted of six strains made up of all members of the blue cluster identified by Chattaway et al. (2019) and a new member not previously tested, namely SRR7516694 (Figure 5A; blue cluster). Eleven strains or half of the second cluster depicted as brown in Figure 5A (total number of strains =22) consisted of five strains from the outbreak cluster 2 (green) cluster and six strains from cluster 3 (red) (Figure 1). Notably, members of the blue cluster (Figure 5A) could be distinguished from the brown cluster and the other strains by a single-nucleotide adenine or guanine (Figure 5B), which occurred in a non-coding region of the prophage genome, devoid of any open reading frames (Supplementary Figure 1).

### Phylogenetic analysis of *Salmonella* Adjame based on the conserved Salmon_SEN34 prophage

**Figure 6:**
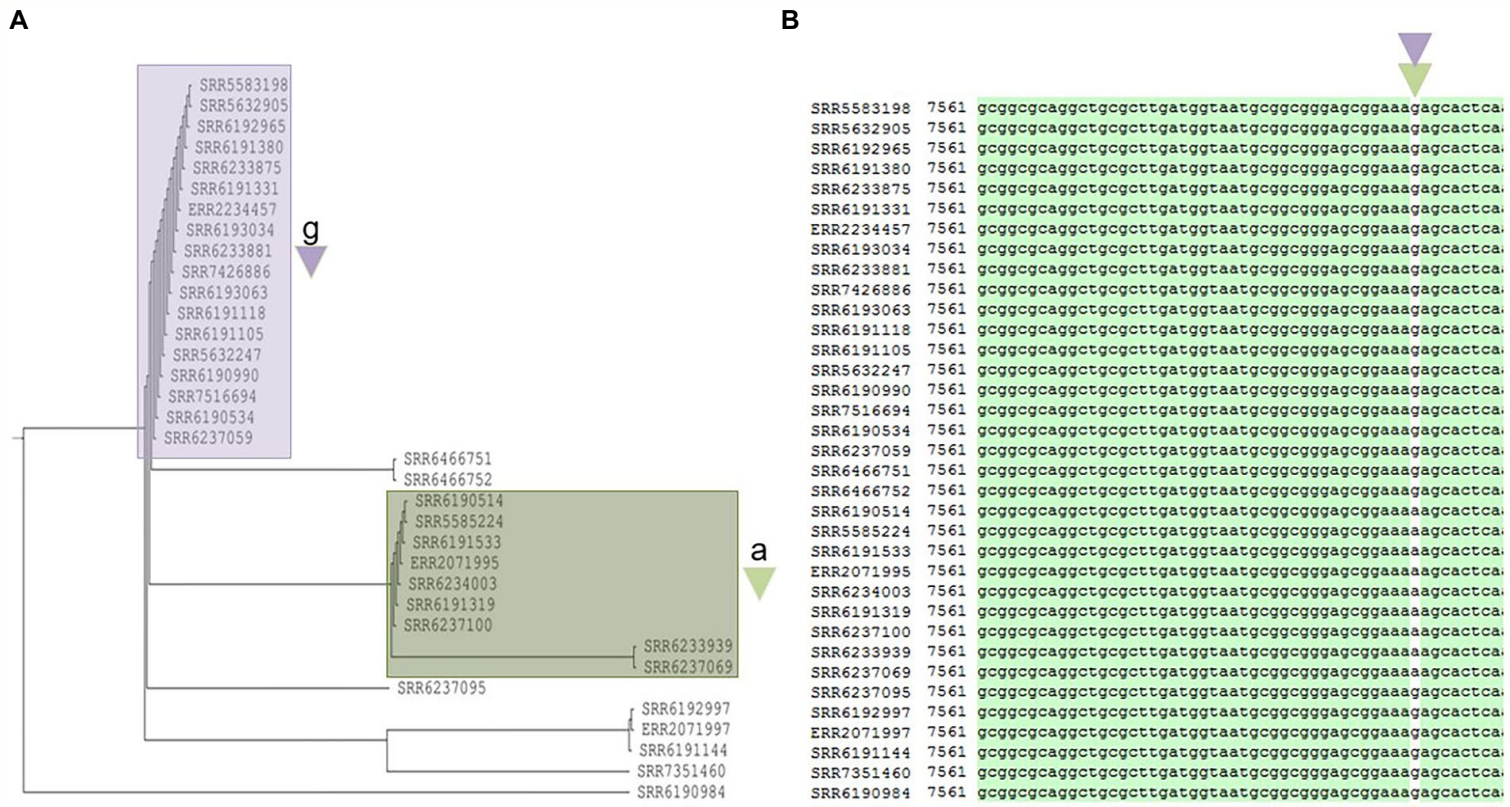
Phylogenetic tree built for 35 *Salmonella* Adjame strains which contains the 2^nd^ most common prophage named Salmon_SEN34 **(A)**. The detected variants are shown in white block **(B)**, with the nucleotide “a” (green triangle) being detected in the green cluster, and the nucleotide “g” (purple triangle) being detected in the purple cluster by merging both the red and blue clusters.

Prophage Salmon_SEN34 was also found to be prevalent in *S.* Adjame and was the next frequently observed phage after Entero_p88, occurring in 35 out of 38 strains. The total length of the aligned sequences of Salmon_SEN34 was 21,185 bp (Supplementary Figure 2A) and a phylogenetic tree developed for the prophage with the Neighbor-Joining method showed two main clusters (Figure 6A). The larger cluster (shown as purple, Figure 6A) consisted of 18 strains and was a mixture of strains obtained before the outbreak (March—April 2017; blue cluster, n = 5), outbreak strains from the red cluster (*n* = 8), and five other strains that were not previously characterized. On the other hand, the smaller group (shown as green) consisted of nine strains which included all the six members of the green outbreak cluster described by Chattaway et al. (2019) including the strain originating from Ireland. Notably, the green group was separated from all the other 26 Adjame strains containing the SEN 34 prophage, whether they were grouped together or not, by a single nucleotide, Guanine or Adenine, found in a hypothetical protein coded for by 183 amino acids (Supplementary Figure 2B). In the green cluster, adenine led to the presence of the amino acid Lysine (AAA), while among the remainders the amino acid Arginine (AGA) was the result of the nucleotide variant Guanine (Figure 6). Thus, a single-nucleotide change in a hypothetical gene of the conserved SEN 34 prophage correlated with the core-genome clustering of the green group of outbreak strains.

### Phylogenetic analysis of *Salmonella* Adjame based on the conserved Burkho_phiE255 prophage

**Figure 7:**
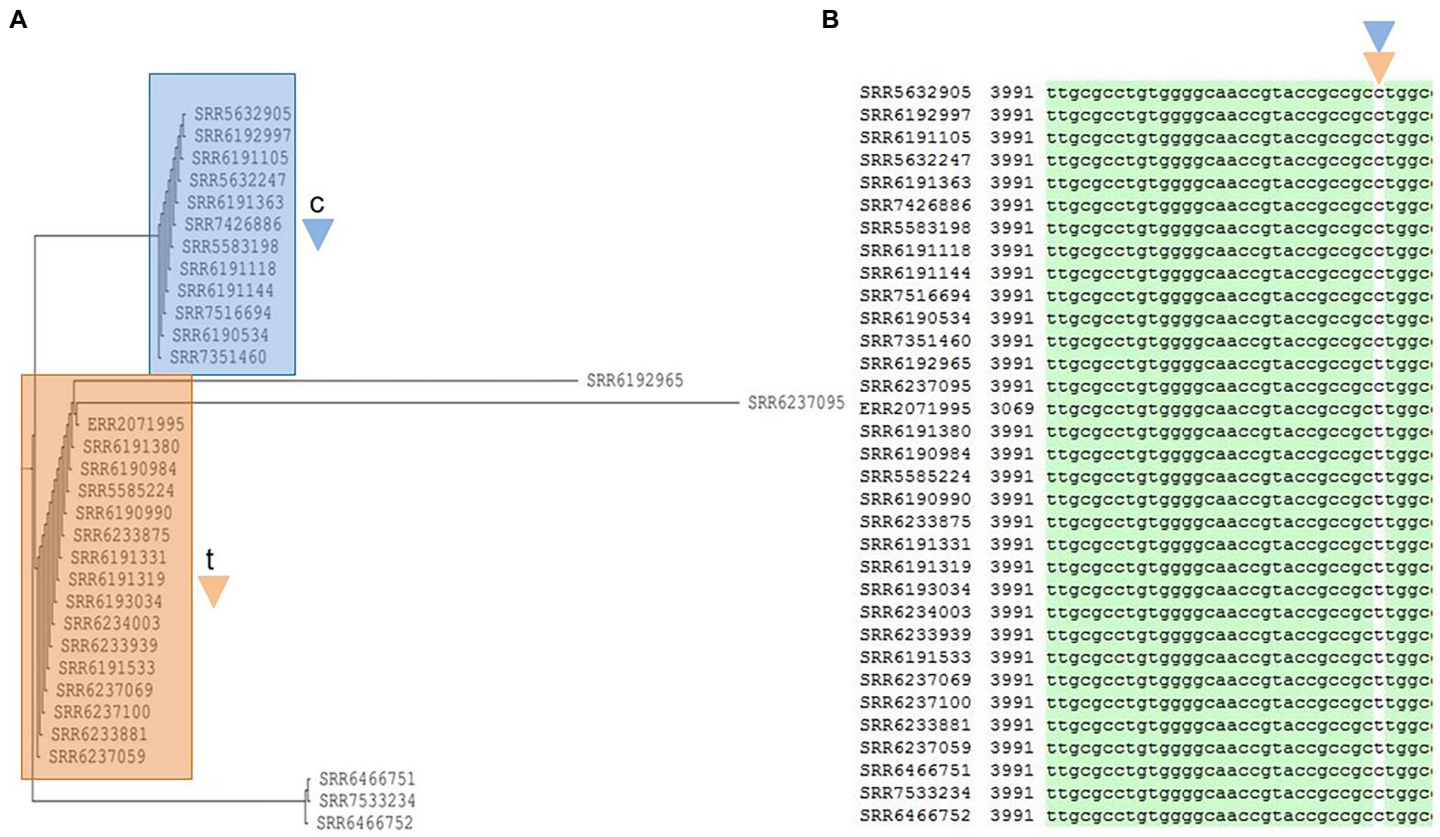
Phylogenetic tree built for 33 *Salmonella* Adjame strains which contains a common prophage named Burkho_phiE255 **(A)**. The detected variants are shown in white block **(B)**, with the nucleotide “c” (blue triangle) being detected in the blue cluster, and the nucleotide “t” (brown triangle) being detected in the brown cluster by merging both the green and red clusters from the June–July 2017 outbreak.

The third most prevalent prophage Burkho_phiE255 was found in 33 out of 38 strains and had a conserved region of 16,423 bp (Supplementary Figure 3A). The Neighbor-Joining phylogenetic tree (Figure 7) showed two major groups which are denoted with blue and brown colors (Figure 7A). The blue cluster consisted of 12 strains, five of which belonged to the previously described blue Cluster 1 by Chattaway et al. (2019). The remaining 7 isolates were similarly excluded from the 2017 outbreak having been identified prior to the event (June 2011 to March 2017; 4 strains) or afterward (November 2017 to October 2018, 3 strains). In contrast, the brown group (Figure 7A) consisted of 12 outbreak strains, i.e., all six members of the outbreak strains that clustered as the green group of Cluster 2, and seven out of eight members of the outbreak strains in the red cluster or Cluster 3. The eighth member of the red cluster lacked the Burkho-phiE255 phage. Notably, the blue cluster (Figure 7A) was separated from the brown cluster and the other strains by a single-nucleotide Cytosine/Thymine, which existed in the open reading frame (ORF) of a phage tail sheath monomer protein with a total of 480 amino acids (Supplementary Figure 3B). In the blue cluster, the variant nucleotide Cytosine was present to form GCC encoding the amino acid Alanine, whereas the Cytosine was replaced with the variant Thymine to form GCT in the brown cluster, but the change was synonymous, and the expression of Alanine (GCT) remained unchanged (Figure 7).

### Phylogenetic analysis of *Salmonella* Adjame based on the conserved Edward_GF prophage

**Figure 8:**
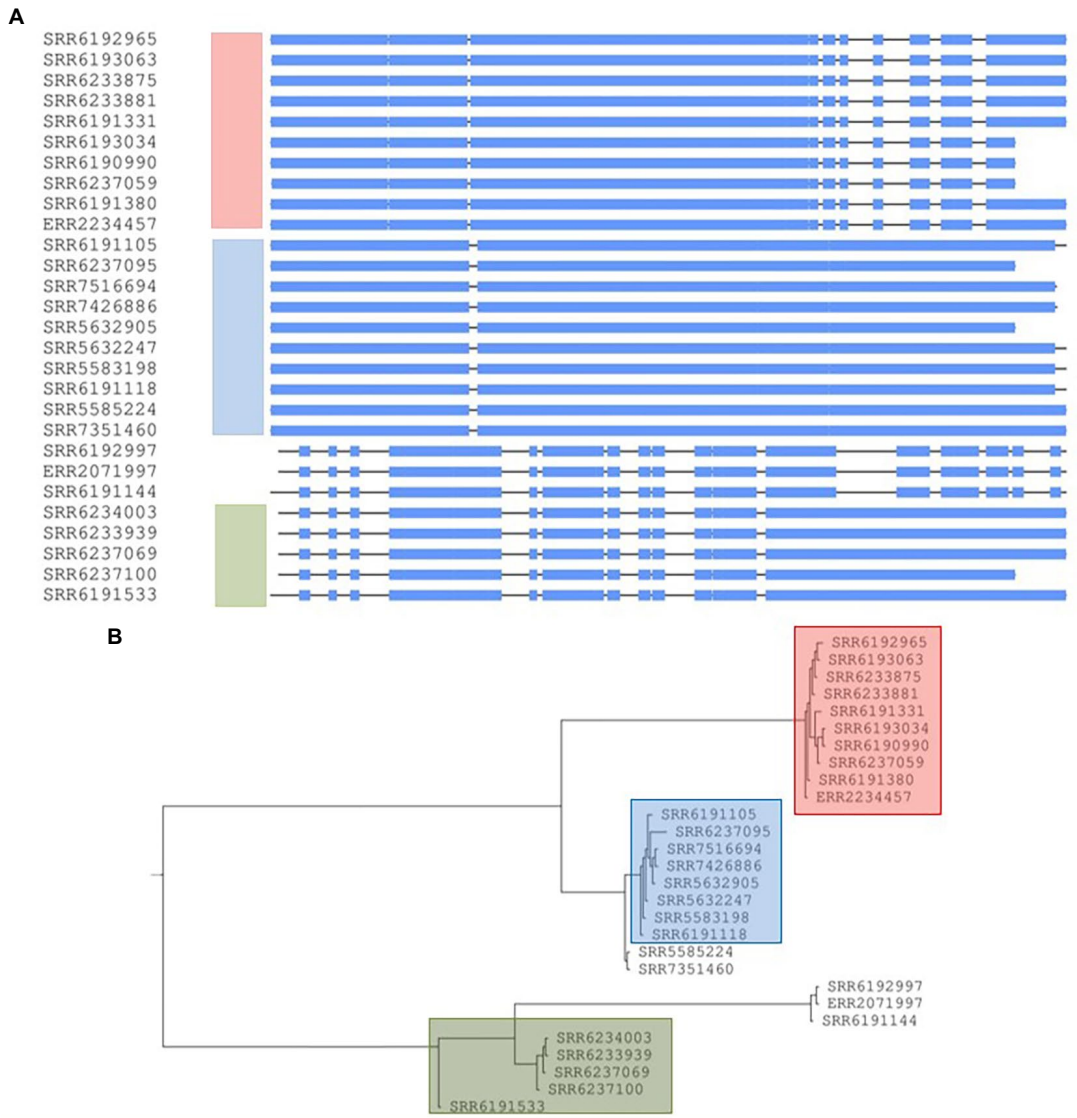
Conserved prophage sequences alignments and phylogenetic tree construction for 28 *Salmonella* Adjame strains based on a common prophage named Edward_GF. **(A)** Nucleotide sequences alignment for this prophage sequences among 28 strains. The three clusters are indicated in three different bars with the color of red, blue and green, respectively. **(B)** Phylogenetic tree built based on the nucleotide alignments. Three different colors represent three different clusters.

The prophage Edward showed a higher sequence variation than the other three prophages described above, namely Entero_p88, Salmon_SEN34, and Burkho_phiE255 (Figure 8A). The aligned sequences of the prophage Edward found in 28 out of the 38 strains (20,234 bp with gaps) generated a phylogenetic tree similar to that produced by core-genome SNP analysis (Figure 8). Seven of the eight strains from the red SNP cluster had the prophage Edward with very similar nucleotide sequence. The only exception is the SRR6190984 which is missing the prophage Edward. On the other hand, one strain (SRR6233875) identified by Chattaway et al. (2019) to be a part of the outbreak but with enough core-genome sequence variation from the remaining outbreak strains in that failed to cluster with the red group (Figure 1; Chattaway et al., 2019) contained a version of the prophage Edward which was indistinguishable from the rest of the red group suggesting a commonality of the source of contamination, despite core-genome heterogeneity. Similarly, one strain (ERR2234457) recovered from Denmark contained a similar prophage Edward and clustered tightly with all members of the outbreak group recovered in England. The Danish strain was recovered 3 weeks ahead of the first isolation in England of strains belonging to the red SNP group and showed some core-genome relatedness but not strong enough to cluster by SNP (Figure 1; Chattaway et al., 2019). The similarity of Edward prophage sequences and proximity of core-genome relatedness suggest that the organisms may have originated from the same or similar sources, and the strains could be inferred to be part of the same outbreak. Out of the seven additional Adjame strains included in this study, which were not previously analyzed by Chattaway et al., 2019, one had an identical prophage Edward sequence with strains belonging to the red SNP cluster, and thereby allowing the strain to be grouped with other Adjame strains. Furthermore, in line with the agreement between the SNP clustering and prophage Edward sequences, all the strains obtained from England between March and April 2017 which were uniform by SNP analysis, also had an identical prophage Edward sequence. The analysis of the prophage Edward sequences shed further light on the relatedness of some strains than did the core-genome analysis alone. In one case, the strain was recovered in March 2017 in England as did many other strains but neither clustered with these other strains by the previous (Chattaway et al., 2019) or current SNP analysis (Figure 1). The prophage Edward sequence also did not generate a cluster with the other strains; however, it revealed some degree of relatedness which shows that the recovery at similar time and prophage sequence may display some niche relationship, and that could not have been inferred by core-genome analysis alone. In conclusion, the grouping based on the prophage Edward sequence largely reproduced the clustering based on core-genome SNPs underscoring the fact that variation in the core genome could be mirrored in the accessory genome.

### Phylogenetic analysis based on the concatenation of conserved sequences of 4 common prophage sequences in 38 strains

**Figure 9:**
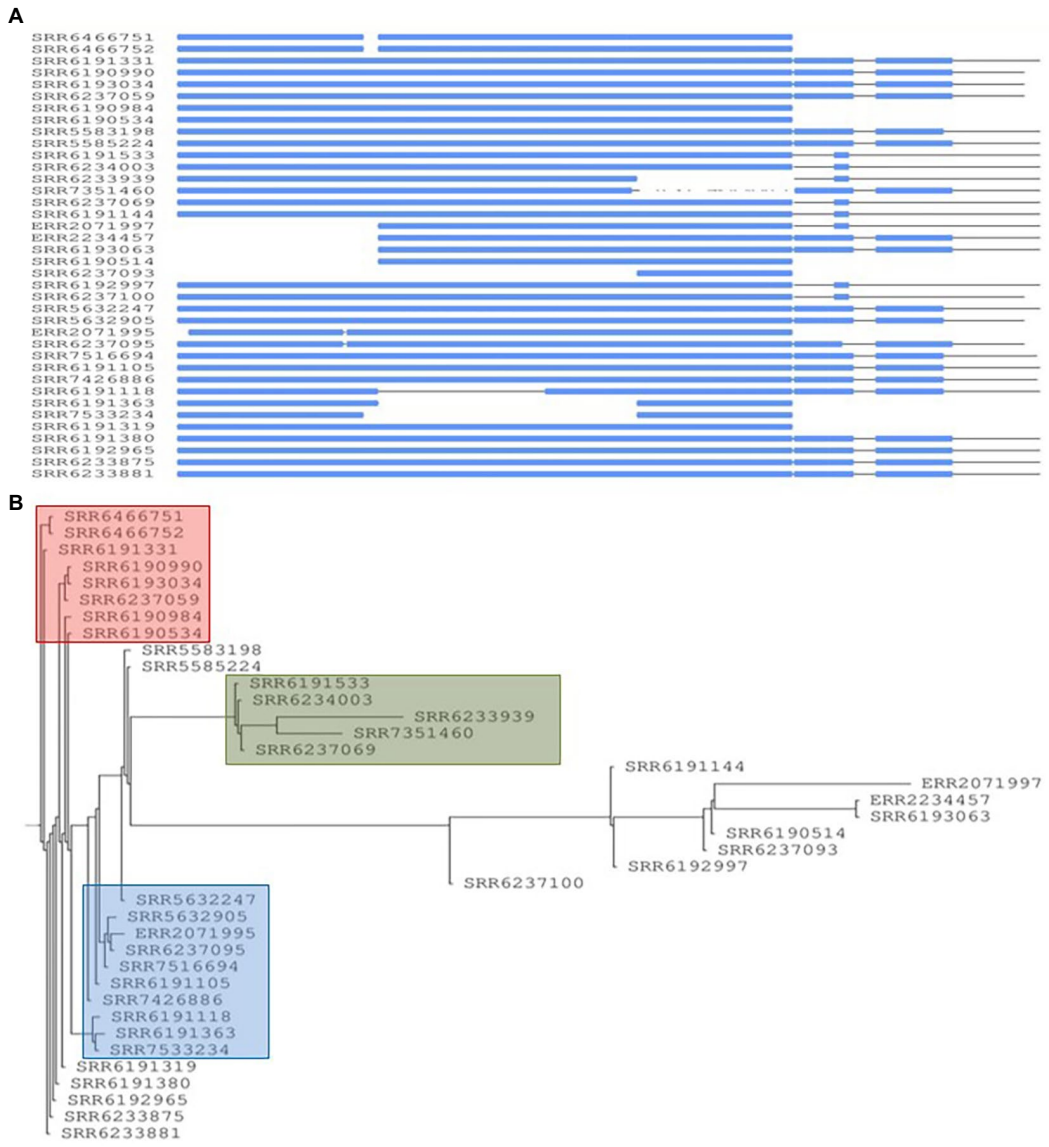
Conserved prophage sequences alignments and phylogenetic tree construction for 38 *Salmonella* Adjame strains based on combining four conserved common prophage named Entero_p88, Salmon_SEN34, Burkho_phiE255 and Edward_GF. **(A)** Nucleotide sequences alignment for these four prophage sequences among 38 strains. The strains lacking of a certain prophage show a blank block and the strains with the similar prophages patterns group together. **(B)** Phylogenetic tree building based on the nucleotide alignments. Three different red, blue, and green colors represent three different clusters.

The concatenation of the four common prophages created an aligned fragment of 70,546 bp (Figure 9A) and the phylogenetic tree showed the presence of the three major groups (blue, green, and red) (Figure 9B) and a topology fairly similar to cluster distribution observed by core-genome analysis, however cluster membership experienced a reduction due to greater diversity resulting from using all four prophages. When compared to the core-genome trees by Chattaway et al. (2019) and in Figure 1, only three of the eight strains in the SNP defined red cluster, three out of six in the green cluster and four out of five in the blue cluster were retained. The analysis of the population structure *S*. Adjame from the lens of the four concatenated prophages showed a greater dispersal when compared to the observation from the core-genome SNP.

### Evolutionary relatedness analysis for identifying the closest serovar with *Salmonella* Adjame

**Figure 10:**
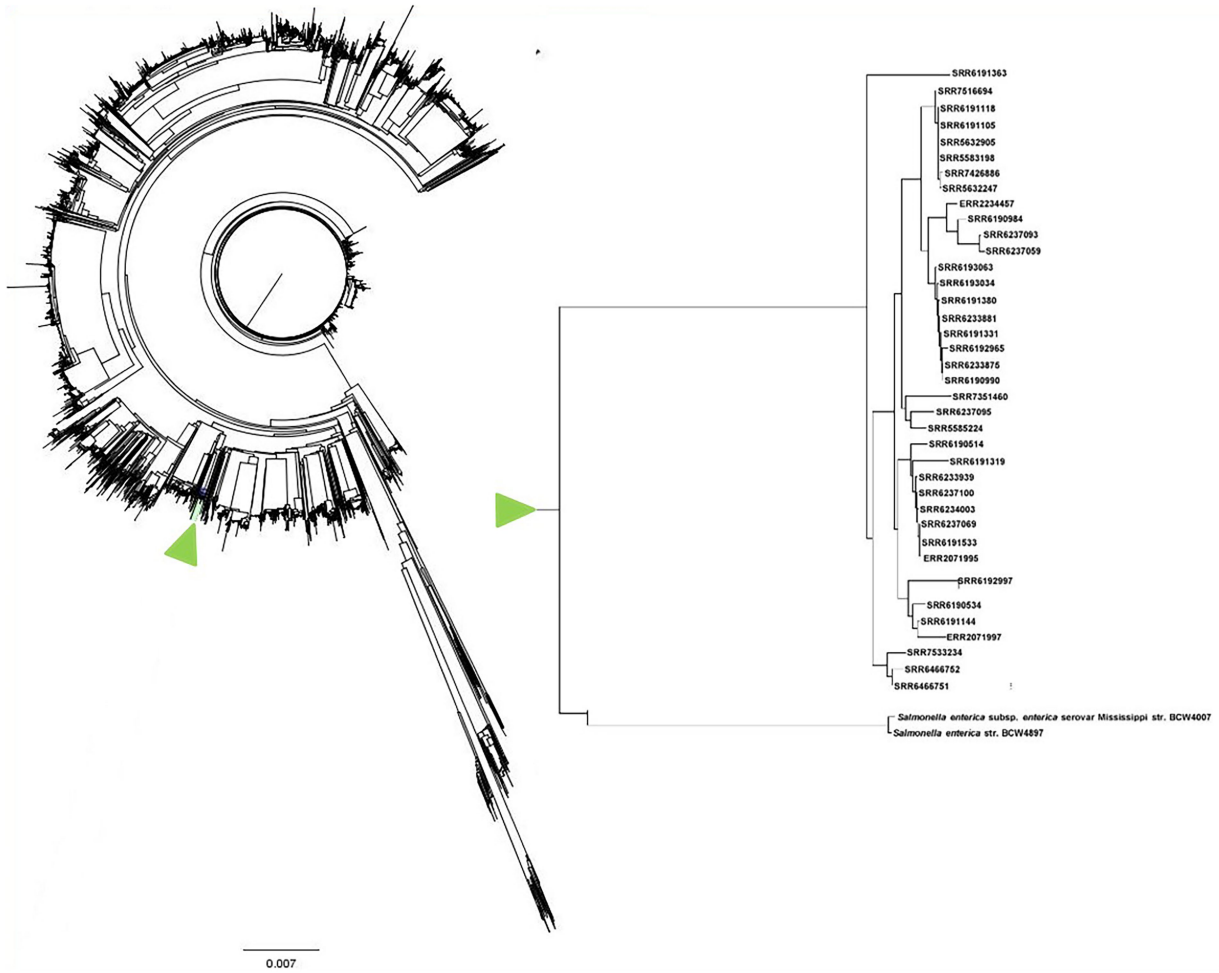
Phylogenetic tree with neighbor-joining genome comparison including a big set of 9,066 RefSeq *Salmonella enterica* genomes and 38 *Salmonella* Adjame analyzed in this study. The branch which contains the 38 studied *S*. Adjame is highlighted in green color. Due to the large number of nodes in this tree, the tip labels are eliminated. The genome branch which contains only the 38 *S*. Adjame strains is enlarged and presented as an inset on the right panel.

A total of 9,066 *Salmonella enterica* genomes obtained from the NCBI database were combined with the 38 *S*. Adjame strains in this study to perform evolutionary relatedness analysis and WGS-based phylogenetic tree construction using an in-house script named “genome_comparator”. This comprehensive genome comparison tree containing the 9,104 strains was built with the Neighbor-joining algorithm and all the *S*. Adjame strains clustered together as expected (Figure 10). Due to the size of the tree and difficulties in displaying all the nodes, the tree is displayed without node tip labeling. For a better visualization, the 38 *S*. Adjame used in this study are highlighted with a green arrow (right panel of Figure 10). The closest *Salmonella* serovar to *S*. Adjame in the phylogenetic tree was the serovar Mississippi (Figure 10).

## Discussion

In this study, we have carried out whole-genome analyses of all 38 strains of the rare *Salmonella* serovar Adjame available in the GenBank at the inception of this study. The strains included those recovered from 14 cases identified in the only known outbreak of *S*. Adjame, which occurred in the summer of 2017 in England. The small size of the entire population of outbreak and sporadic strains provided an excellent cohort to dissect the variability between the core and accessory genome of the strains and to overlay the analytical inferences on the epidemiology of *S*. Adjame, especially regarding the time of transmission to individuals. Analysis of the 2017 outbreak by Chattaway et al. (2019) using the Snapper DB high-quality SNP pipeline and EnteroBase *Salmonella* core-genome multilocus sequence typing scheme showed that the clinical cases were due to heterogeneous strains of *S*. Adjame rather than a point source exposure. In this study, our goal was to carry out a comparative analysis of the core and accessory genomes of the organisms to evaluate the extent of the heterogeneity of organisms involved in the outbreak, compare the outbreak strains with the sporadic strains including those isolated around the time of the outbreak, in order to provide an insight into the population structure of the rare *Salmonella* organism. At the onset, we evaluated the variation in the core genome of the Adjame strains by means of two SNP analyses, namely Parsnp and kSNP, and observed a clustering of the outbreak strains similar to that reported by Chattaway et al. (2019). Our results confirmed a considerable genetic variation among the strains identified as part of the outbreak. Our Parsnp analysis (Figure 1) showed that organisms identified as part of the 2017 outbreak (green and red cluster) had a closer ancestry when compared to all the other strains analyzed including organisms belonging to the blue cluster, which is in contrast with the observations by Chattaway et al. (2019). These results indicate that different SNP analysis algorithm or analysis of the core genome may give different clustering outputs, and additional information may be required from further analysis of the genome to clarify strain relatedness. To that end, the accessory genome may shed the required light to evaluate the characteristic of the population including relatedness of the strains and provide evidence-based inferences about evolutionary changes in the population. Prophage genes form the majority of the bacterial accessory genomes and have great potential to be used as highly discriminatory markers for *Salmonella* (Mottawea et al., 2018) and *Listeria monocytogenes* (Zamudio et al., 2020). In this study, we analyzed the prophage sequences in the *S*. Adjame strains previously reported as part of the atypical outbreak (June—July 2017), and of a group of strains recovered just prior to the outbreak, mainly between March and April 2017), as well as other sporadic strains recovered as early as October 2008 or as late as December 2018.

The pronounced diversity in the prophage genomes among our collection of *S*. Adjame strains verified and reinforced the heterogeneity of the organisms recovered from the atypical outbreak (Figures 3, 4) Twenty-four different prophages were detected while only four of them were present in the majority of the studied strains. Upon further dissection of the individual prophage sequence profile, we found that one nucleotide variant in each of the conserved prophage region seemed sufficiently informative for assigning certain strains to a cluster (Figures 6–8). For instance, the Adenine/Guanine base in the conserved regions of prophage Entero_p88 (Figure 5) and the Cytosine/Thymine base in the conserved regions of the prophage Burkho_phiE255 (Figure 7) were used to separate the March 2017 sporadic strains from the rest of the strains recovered during the period spanning the outbreak, i.e., June to July 2017, highlighting the potential use of a single nucleotide in the accessory genome as a marker to differentiate between sporadic and outbreak strains. Notably, the detected Adenine/Guanine location in the prophage Entero_p88 was not included in any of the predicted ORF, which showed that mutations in a non-coding region could provide adequate information in strain characterization. Other authors have demonstrated the potential role of non-coding regions of *Salmonella* in pathogenesis in animals and virulence (Gong et al., 2011; Hammarlof et al., 2018). For prophages that showed greater variability within the conserved regions such as prophage Edward (Figure 8A), three different prophage patterns were observed among the 28 strains that harbored the prophage, and these translated into three distinct clusters in the phylogenetic tree (Figure 8). The clustering pattern observed in the phylogenetic tree developed for the prophage Edward sequence (Figure 8) was similar to the SNP analysis clustering using the SnapperDB algorithm (Chattaway et al., 2019). Thus, the inclusion of additional prophages in our analysis predictably led to a demonstration of an even greater heterogeneity among the Adjame strains. Indeed, when we combined the four conserved prophage sequences to generate a phylogenetic tree and overlaid the groups with the previously described cluster designations (i.e., red, green and blue clusters, Figure 9), we were able to clearly show an even greater degree of heterogeneity, which became even enhanced in the phylogenetic tree produced using all 24 different prophages (Figure 4). We have used the PST to show that highly related *Salmonella* are indistinguishable from one another (Ogunremi et al., 2014); however in this study, only a few Adjame strains appear to be very closely related (e.g., SRR6466751 and SRR6466752). We propose that the 2-year time differences between these strains (2011 and 2013) may have led to mutational changes detectable by the PST since the assay is affected by nucleotide variation in a target prophage if the assay parameters are set at a high stringency as we have done. Our study showed that prophage sequence analysis provided enough granularity to distinguish all the strains of Adjame in the population from one another. It is possible that the biology of the organism or the niche environment occupied by the strains predisposed them to one or all of the following: receptivity of the organisms to phages, integration into the genomes, and rapid changes in the chromosomal integrated bacteriophages. Prophages are known to represent a diverse arsenal that allows bacteria to adapt to their ever-changing and competitive environments (Bobay and Ochman, 2017), and the diversity within and among the many prophages observed in this study is supportive of this claim. However, we also noticed limited size variation in two prophages namely, the prevalent Burkho_phiE255 (35 of 38 strains) and the less prevalent Entero-cdtl (19 out of 38 strains). We speculate that the high conservation seen in these prophages suggests that they may not be a factor in driving the diversity of Adjame. It will be interesting to see if the size conservation of the prophages has arisen from their low susceptibility to recombination with other phages.

We noticed that the outbreak strains obtained during the month of June 2017 and characterized by SNP analysis as the green cluster showed the most closely related prophage profiles (Figure 4). Another isolate not identified as part of the 2017 outbreak, namely, SRR6237069, but nevertheless recovered during the same time as the outbreak (June 2017) showed relatedness in profile prophage with the other green isolates as defined by SNP (Figures 1, 2) and prophage analysis (Figure 4). Furthermore, the four prevalent prophages were present in SRR6237069 and segregated as did the rest of the green isolates. The prophage analysis also placed the ERR2071995 isolate obtained from Ireland and grouped by SNP analysis with the green cluster (Chattaway et al., 2019; Figures 1, 2, 6). The additional observation obtained here from application of the highly discriminatory prophage typing would therefore suggest that the outbreak was international in scope based on close genetic relatedness of the isolates both at the core and prophage genome levels.

One of the isolates from the green cluster, SRR6233939, showed a similar prophage profile to another isolate from the red cluster. This green isolate was exceptional in that it was the only one missing the Entero_p88 isolate and therefore occupied the most distant position among the green group by prophage analysis. Along the same line, SRR6192965, which has a core genome that grouped as a red cluster showed noticeable variation in the prophage profile as indicated by a long branching of the prevalent Burkho_phiE255 prophage (Figure 7). This, and other prophage changes led to a dissociation of SRR6192965 from the other strains that clustered into the red group when all four prevalent phages were analyzed (Figure 9). The prophage analysis showed a demarcation of the outbreak strains that were grouped together into the red cluster based on the core genome into two groups and two singletons, indicating an even greater heterogeneity than previously described by Chattaway et al. (2019). The variation seen among the prevalent prophages may shed light into the commonality among strains, provide evidence of the substructures within the population, and enable the tracking of closely related strains (e.g., green isolates from England and Ireland).

Two strains obtained during the course of outbreak in June 2017 (SRR6190984) and July 2017 (SRR6237093) as well as a third strain obtained months after the outbreak in March 2018 (SRR7351460) were unique in their prophage profile and did not show relatedness with any other isolate (Figure 4). Only one of these strains was grouped as part of the outbreak by clustering with the red group (SRR6190984). While this strain is scored as part of the red cluster based on core-genome level, it is very different from the others at the prophage level. Indeed, analysis of the prophage Entero_p88 in this strain revealed a strong similarity with another group of five, non-outbreak strains obtained between October 2008 and June 2017 (Figure 5) and these five strains formed a loose cluster at the core-genome level according to Chattaway et al. (Figure 5). Thus, it is remarkable that a strain involved in the England outbreak (SRR6190984) and which clustered with other outbreak strains (red cluster) could have at some point acquired a version of the Entero_p88 prophage or mutations that are different from those by other members in the cluster, but similar to another version of the Entero_p88 seen in another group with a different core-genome attribute. This may suggest that although the “genetic backbone” of the SRR61090984 as defined by the core genome is related to the other outbreak strains, it did acquire accessory genomic attributes by occupying a similar niche with the five additional strains that were not involved in the outbreak. In that sense, the evaluation of the core- and accessory-genome properties of the strains helped to show lineage similarities regardless of the time of recovery from patients. Thus, while the atypical outbreak was defined by the time the patients presented with symptoms using valid epidemiological parameters (i.e., between June to July 2017), there were strains obtained during and outside the time period, by a combination of core-genome and accessory-genome analysis, but not the former alone, that demonstrably and convincingly displayed close relatedness. Prophage level relatedness could therefore point to a shared history of strains (e.g., niche) even when there is little similarity at the core-genome level.

For such a rare serovar, we were interested in identifying the closest genetic serovar relatives. Our Neighbor-joining phylogenetic tree consisting of all 38 *S*. Adjame strains and 9,066 Refseq *Salmonella enterica* genomes from the NCBI RefSeq database (Figure 10), showed that the closest serovar to *S*. Adjame was *S.* Mississippi. Apart from the closer relatedness shown by WGS-based analyses, these two serovars also shared some somatic O (O) antigen and Flagella H phase 2 (H2) antigen, i.e., *S*. Adjame with O (13, 23) and H2 (1, 6) and as *S.* Mississippi with O (1, 13, 23) and H2 (1, 5), respectively. In contrast to *S*. Adjame, the serovar Mississippi has been detected from many parts of the world and from various sources including humans, animals, and environmental sources (Ashbolt and Kirk, 2006; Ford et al., 2019).

Although the prophage genome is very distinct from the core genome as measured by GC content, which is further accentuated by the unique biology of the phage virus, e.g., high recombination, evolutionary changes in the core genome can be matched to changes in the prophage genome suggesting a common but unidentified signaling mechanism contributing to a synchronized evolutionary change in the disparate parts of the bacterial chromosome. The detection of synchronized changes in both genomic fractions, core and accessory, may indicate a considerable period of co-existence in the strain. The observations in this study of identical single-nucleotide mutations in prophages (Entero_p88 and Burkho_phiE25) belonging to the green and red clusters appear to support our inference of the cluster relatedness however the contrast is observed for the third common prophage (Salmon_SEN34) in which identical SNP was observed in the red and blue clusters, indicating that caution may be warranted not to over-interpret the mutational changes in the prophages as they may not always infer a direct relationship.

In conclusion, this study explored the diversity of prophages, the main component of the accessory genomes in *Salmonella*. Our results indicate that *S*. Adjame prophages collectively displayed a considerable degree of variation that allowed each and every strain in our cohort to be characterized at a very high resolution in a manner that allowed each to be distinguished from one another. Surprisingly, the pattern of variation observed in one of the most prevalent prophages, Edward_GF, accurately reproduced the clustering pattern seen by core-genome analysis, indicating that correlative mutational changes may be found in the core and accessory genomes. The routine use of core-genome analysis for characterizing *Salmonella* stands to benefit from the application of our highly discriminatory PST pipeline to develop a very comprehensive definition of genomic attributes and identify similarities or dissimilarities among isolates. Prophages as well as their patterns of variation can shed light on strain relatedness and niche characteristics even when these changes are not imprinted on the core genome.

## Affiliations

MAC is affiliated to the National Institute for Health Research Health Protection Research Unit (NIHR HPRU) in Genomics and Enabling Data at University of Warwick in partnership with the UK Health Security Agency (UKHSA). She is based at UKHSA. The views expressed are those of the author(s) and not necessarily those of the NIHR, the Department of Health and Social Care or the UK Health Security Agency. LG is the Leung Family Professor of Food Safety, Department of Food Science, University of Guelph, Guelph, Canada. DO is an Adjunct Professor, Department of Food Science, University of Guelph., Guelph Canada. RG is currently employed at the National Microbiology Laboratory, Public Health Agency of Canada.

## Data availability statement

The original contributions presented in the study are included in the article/Supplementary material, further inquiries can be directed to the corresponding author. Raw read genome sequences are available in the Short Read Archivs of the National Centre for Biotechnology Information (NCBI) database using the accension number and SRA identification provided in Table 1.

## Author contributions

RG analyzed the core-genome and prophage sequences, prepared the figures and tables, and drafted the manuscript. M-OD analyzed the prophages and wrote the scripts. MC participated in study design, provided background data, and reviewed manuscript. LG contributed to study conception and reviewed the manuscript. DO conceived the study and participated in the data analysis, and in manuscript development and review. All the authors revised and approved the manuscript.

## Funding

This study was funded by grants from Genome Canada to the *Salmonella* Syst-OMICS project, Genome Research and Development Initiative of the Government of Canada, Canadian Security and Science Program of the Department of National Defense, and the Canadian Food Inspection Agency.

## Conflict of interest

The authors declare that the research was conducted in the absence of any commercial or financial relationships that could be construed as a potential conflict of interest.

## Publisher’s note

All claims expressed in this article are solely those of the authors and do not necessarily represent those of their affiliated organizations, or those of the publisher, the editors and the reviewers. Any product that may be evaluated in this article, or claim that may be made by its manufacturer, is not guaranteed or endorsed by the publisher.

## Footnotes

^1^http://enterobase.warwick.ac.uk/

^2^https://github.com/duceppemo/genome_comparator
