# Supplementary figures and images for: Application of prophage sequence analysis to investigate a disease outbreak involving Salmonella Adjame, a rare serovar and implications for the population structure
Source: Front Microbiol. 2023 Mar 3;14:1086198. doi: 10.3389/fmicb.2023.1086198 (PMC10020630; doi:10.3389/fmicb.2023.1086198)

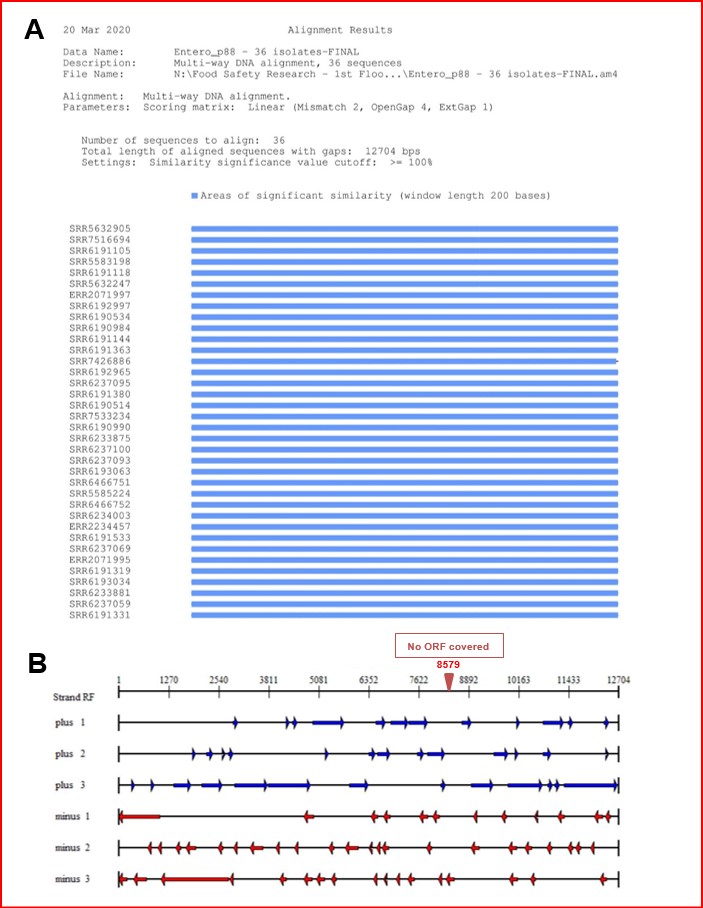

Supplement: Supplementary file 2 [file Image_1.JPEG]

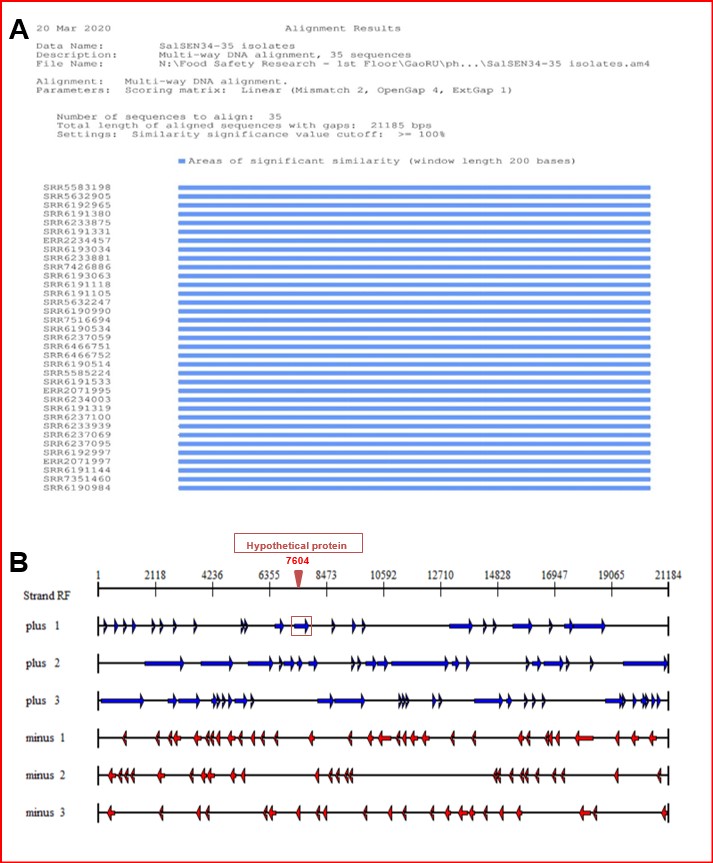

Supplement: Supplementary file 3 [file Image_2.JPEG]

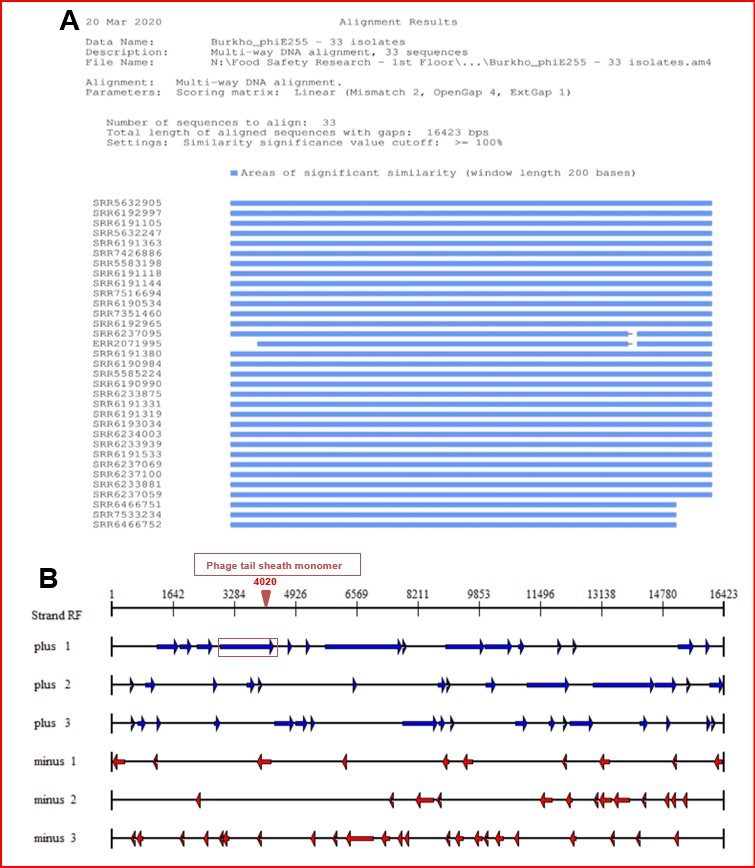

Supplement: Supplementary file 4 [file Image_3.JPEG]
